# Correction: Distribution and survival strategies of endemic and cosmopolitan diazotrophs in the Arctic Ocean

**DOI:** 10.1038/s41396-023-01444-7

**Published:** 2023-06-01

**Authors:** Takuhei Shiozaki, Yosuke Nishimura, Susumu Yoshizawa, Hideto Takami, Koji Hamasaki, Amane Fujiwara, Shigeto Nishino, Naomi Harada

**Affiliations:** 1 Atmosphere and Ocean Research Institute, The University of Tokyo, Kashiwa, 277-8564 Japan; 2 Research Centre for Bioscience and Nanoscience, Japan Agency for Marine-Earth Science and Technology (JAMSTEC), Yokosuka, 237-0061 Japan; 3 Center for Mathematical Science and Advanced Technology, JAMSTEC, Yokohama, 236-0001 Japan; 4 Department of Integrated Biosciences, Graduate School of Frontier Sciences, The University of Tokyo, Kashiwa, 277-8564 Japan; 5 Collaborative Research Institute for Innovative Microbiology, The University of Tokyo, Bunkyo-ku, 113-8657 Japan; 6 Research Institute for Global Change, JAMSTEC, Yokosuka, 237-0061 Japan

**Keywords:** Microbial biooceanography, Environmental sciences

Correction to: *The ISME Journal* 10.1038/s41396-023-01424-x, published online 23 May 2023

In this article the author name Takuhei Shiozaki was incorrectly written as Shiozaki Takuhei.

The original article has been corrected.

