# Supplementary material for: Distribution and survival strategies of endemic and cosmopolitan diazotrophs in the Arctic Ocean
Source: ISME J. 2023 May 23;17(8):1340–50. doi: 10.1038/s41396-023-01424-x (PMC10356936; doi:10.1038/s41396-023-01424-x)
Supplement: Supplementary file 1 — Supplementary Infomation [file 41396_2023_1424_MOESM1_ESM.docx]

Supplementary Information for

Distribution and survival strategies of endemic and cosmopolitan diazotrophs in the Arctic Ocean

T. Shiozaki, Y. Nishimura, S. Yoshizawa, H. Takami, K. Hamasaki, A. Fujiwara, S. Nishino, N. Harada

Correspondence to: shiozaki@g.ecc.u-tokyo.ac.jp

Fig. S1 Codon usage of Arctic and other diazotrophs. Principal component analysis performed based on the frequency of 64 types of codons used in the total proteome of each genome.


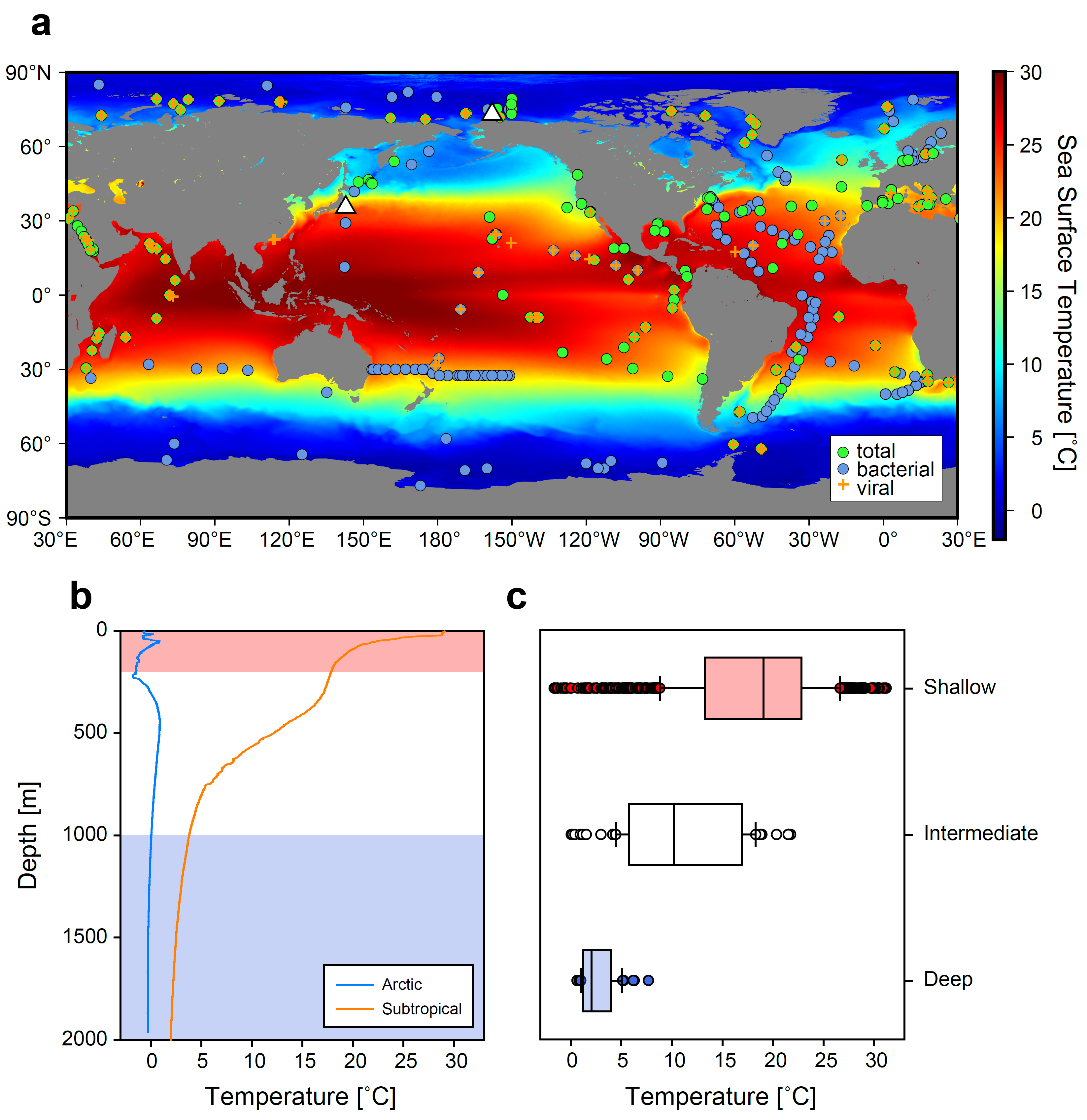


Fig. S2 Geographic distribution of the metagenomes analyzed and their water temperatures. a. Locations where samples for metagenomic analysis were collected. Green and blue circles and orange crosses indicate the locations where samples for total, bacterial, and viral fractions were collected, respectively. Background contours indicate sea surface temperature composited across the entire mission of Aqua-MODIS (https://oceancolor.gsfc.nasa.gov/). b. Temperature profile of the Arctic Ocean and subtropical ocean (white triangle in a) determined during the R/V Mirai MR20-05C cruise (https://www.jamstec.go.jp/iace/e/report/). Red, white, and blue lines indicate shallow (≤200 m), intermediate (200–1000 m), and deep (≥1000 m) layers, respectively. c. Box plot of temperature at the depths from which the metagenomic samples were collected in each layer. The line within the box indicates the median, and the upper and lower limits of each box indicate the 25th and 75th percentiles, respectively. The error bars indicate the 10th and 90th percentiles. Data beyond the error bars are plotted individually.


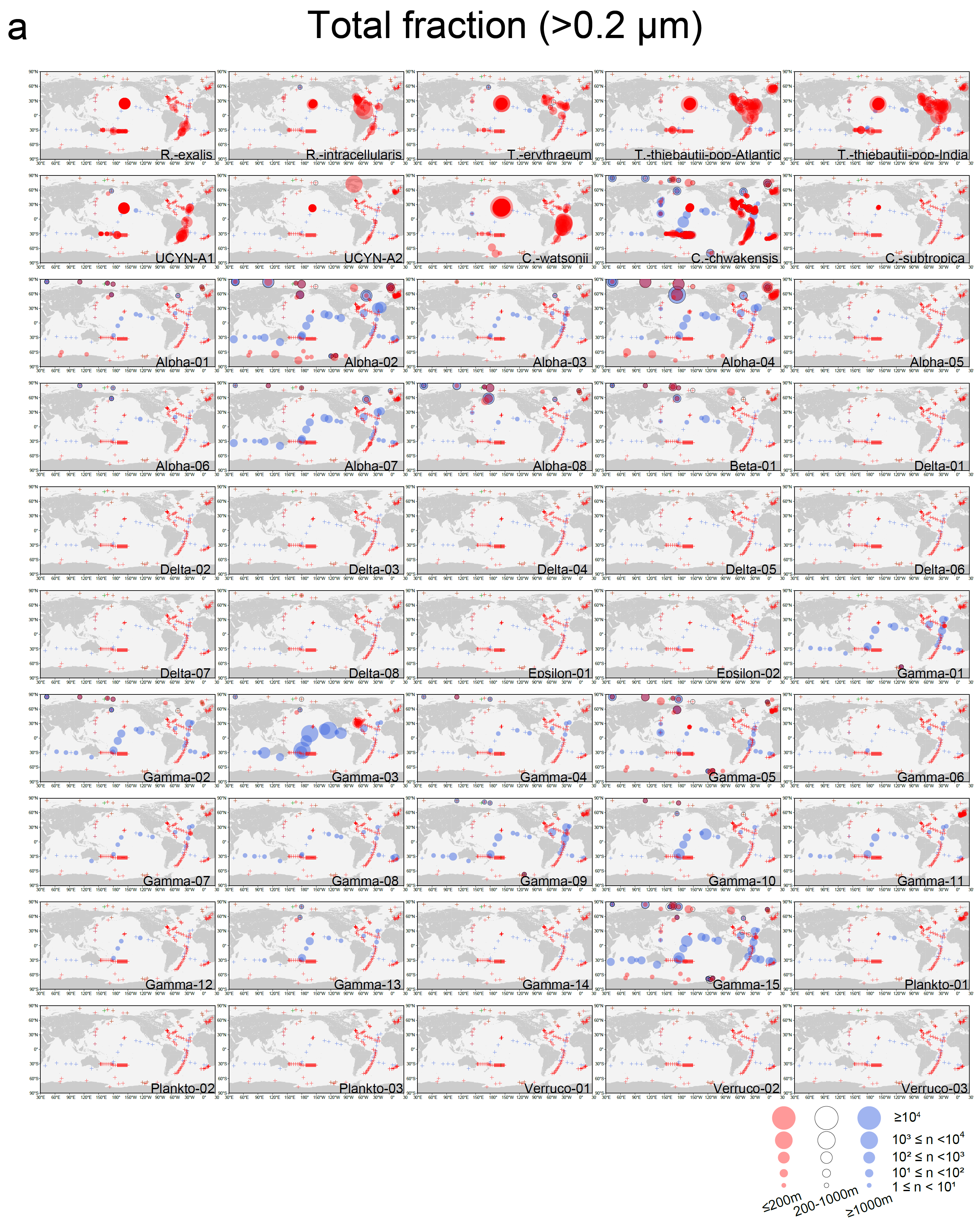


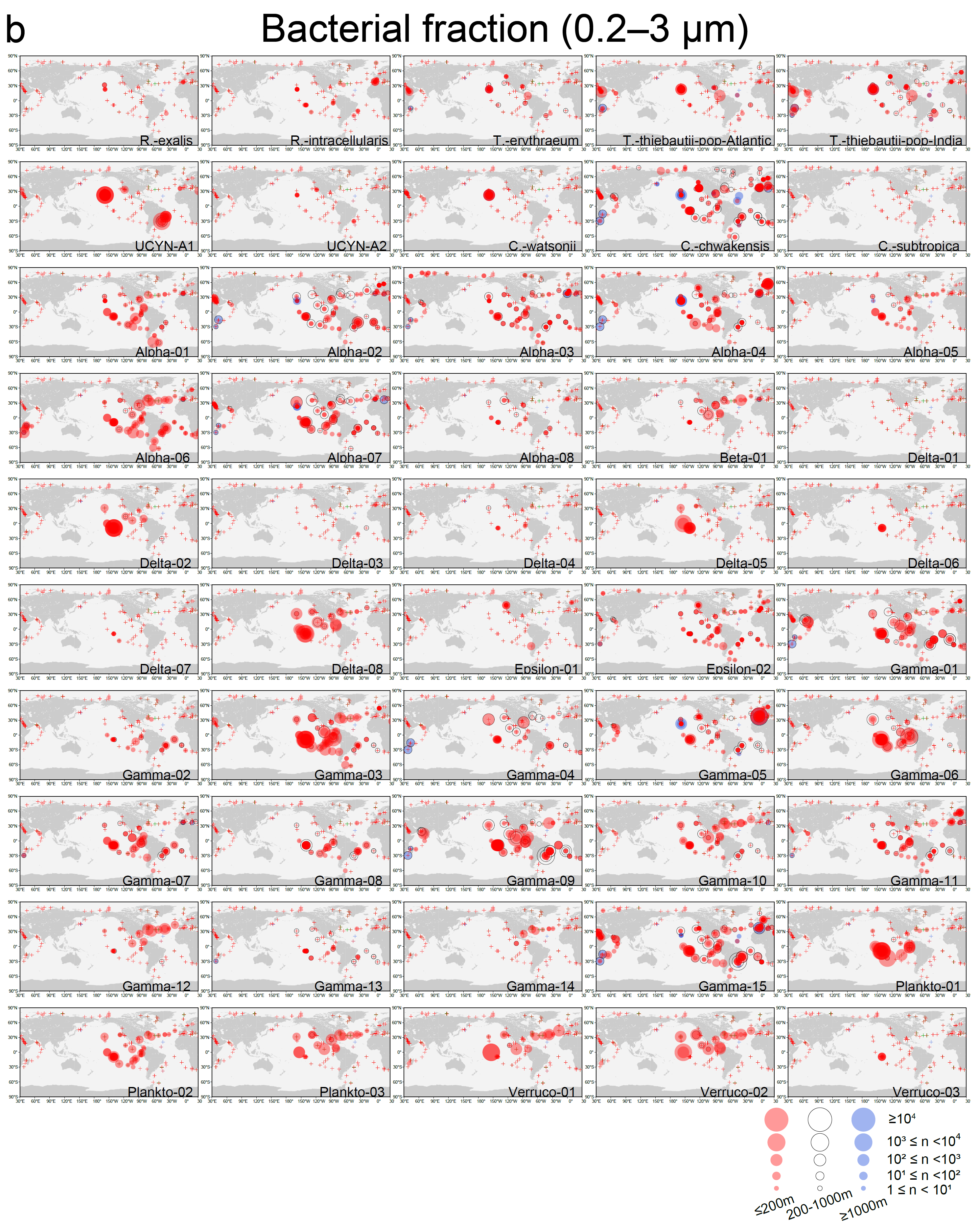


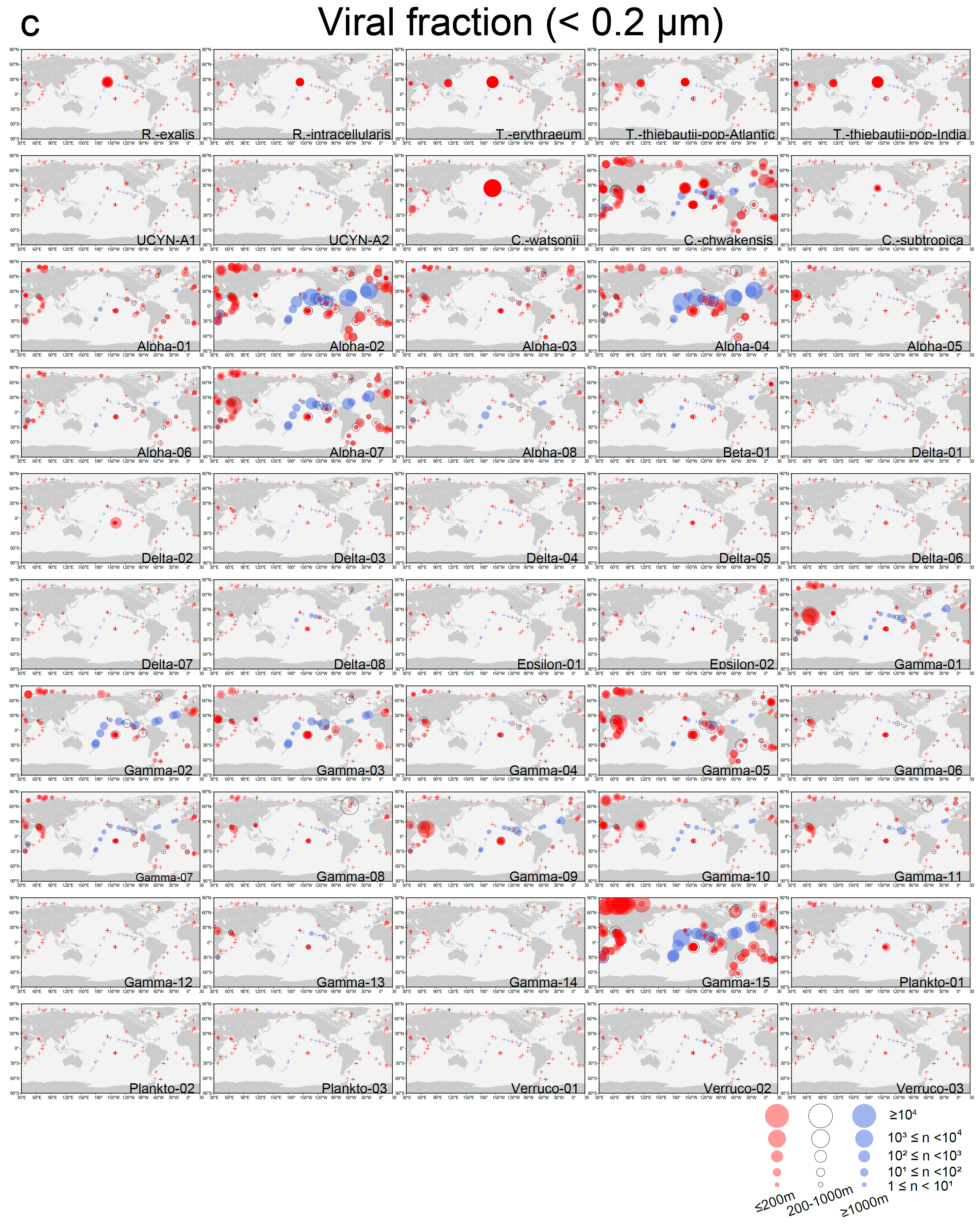


Fig. S3 Depth-resolved abundance of known marine diazotroph genomes in global ocean for each size fraction (a, total; b, bacterial; and c, viral fraction) for each layer (≤200 m, red; 200–1000 m, black open circle; and ≥1000 m, blue). The area of each circle is proportional to the abundance it represents. The plus signs indicate the location with CPMM lower than 1.

Fig. S4 The proportion of seven amino acids (I, V, Y, W, R, E, L) in the total proteome of each genome. The line within the box indicates the median, and the upper and lower limits of each box indicate the 25th and 75th percentiles, respectively. Whiskers are extended to the furthest point within 1.5 times of the interquartile range from the lower or upper quartile. All data points are plotted individually.

Fig. S5 The pangenome of UCYN-A visualized with the anvi’o [37]. The location from which each genome was retrieved and other details are summarized in Table S2. The dark green bar indicates genes shared by all genomes in this analysis. The ANI matrix was generated using the “anvi-compute-genome-similarity” command in anvi’o.

**Supplemental Table**

Table S1 Summary of metagenome data used in this study. This table contains various sample metadata including metagenome accession number, sampling location, and filtration fraction.

Table S2 Summary of UCYN-A genomes used in this study. This table contains genome quality, retrieved location, reference, and *nifH* sequences.

Table S3 Summary of diazotroph genomes used in this study. The table contains genome statistics, genome quality, genome-based taxonomy, minimum generation time, number of genes that encode cold-inducible proteins and glycosyltransferases, and usage of codons and amino acids.

Table S4 Primers and TaqMan probes for qPCR of *nifH*.

Table S5 The *nifH* sequence of Arctic diazotroph MAGs and its compatibility with *nifH* primers. a. The *nifH* sequence of Arctic diazotroph MAGs. b. Compatibility between *nifH* primers and *nifH* of Arctic diazotroph MAGs.

Table S6 Summary of diazotroph genome abundance (count per million microbe genomes of a total community, CPMM) in the total, bacterial, and viral fraction.

Table S7 List of the functional module completion ratio (MCR) and its Q-value as calculated by Genomaple for each diazotroph genome.

Table S8 Summary of environmental variables and abundance of diazotrophs in the western Arctic Ocean during late summer 2015, 2016, and 2017.
